# Draft Genome Sequence of Paucibacter aquatile CR182^T^, a Strain with Antimicrobial Activity Isolated from Freshwater of Nakdong River in South Korea

**DOI:** 10.1128/genomeA.00194-18

**Published:** 2018-04-26

**Authors:** Eu Jin Chung, Gang-Guk Choi, Young-Ho Nam, Ahyoung Choi

**Affiliations:** a Freshwater Bioresources Culture Research Bureau, Nakdonggang National Institute of Biological Resources (NNIBR), Sangju, South Korea

## Abstract

This report details a draft genome sequence of Paucibacter aquatile CR182^T^, isolated from river water, which contains 5,523,543 bp, has a G+C content of 66.3%, and harbors 4,544 protein-coding genes in 4 contigs. These genome data provide insights into the genetic basis of this strain’s antibacterial activity and adaptive mechanisms.

## GENOME ANNOUNCEMENT

The genus Paucibacter was first proposed in 2005 (1) with the type species Paucibacter toxinivorans, which belongs to the family Comamonadaceae in the phylum Betaproteobacteria. Members of the genus Paucibacter characteristically are Gram stain negative, rod shaped, and often resistant to antibiotics or toxic compounds (1–3). Here, we report the draft genome sequence of Paucibacter aquatile CR182^T^, isolated from freshwater of the Nakdong River, South Korea, during a screen for bacteria with antimicrobial activity (3). Strain CR182^T^ has been deposited in the Korean Culture Center of Microorganisms (KCCM) and the Nite Biological Resource Center (NBRC) under the accession numbers KCCM 9028 and NBRC 113032, respectively.

Genomic DNA was extracted using a genomic DNA purification kit (Promega). A 20-kb SMRTbell genome library was sequenced using the PacBio RS II single-molecule real-time (SMRT) sequencing platform at ChunLab (Seoul, South Korea). The bacterial genome was assembled *de novo* into four contigs, with an average genome coverage of 230.9×, using the PacBio SMRT Portal (2.3.0) and the Hierarchical Genome Assembly Process (HGAP) (4). The Prokaryotic Genome Annotation System (Prokka) pipeline was used for genome annotation (5). The data were submitted to the Rapid Annotations using Subsystem Technology (RAST) server (6) and the National Center for Biotechnology Information (NBCI) genome sequence database. We searched for potential coding sequences using the Basic Local Alignment Search Tool (BLAST) against the UniProt (7), Pfam (8), and Clusters of Orthologous Groups of proteins (COG) (9) databases. Signal peptides and transmembrane helices were predicted using SignalP 4.1 (10) and TMHMM v2.0 (11). rRNA, tRNA, and other miscellaneous features were predicted using RNAmmer v1.2 (12), tRNAscan-SE v1.21 (13), and Rfam v12.0 (14).

The draft genome sequence of P. aquatile CR182^T^ is 5,523,543 bp, with a G+C content of 66.3%. The genome is predicted to have 4,544 coding sequences (CDSs), 72 tRNA genes, and 28 rRNA genes. We identified various antibacterial genes in the genome of P. aquatile CR182^T^; antiSMASH shell analysis (15) revealed that the genome harbors 9 gene clusters involved in the biosynthesis of lantipeptide, hserlactone, bacteriocin, terpene, and a nonribosomal peptide synthetase (NRPS), a kind of amychelin. Analysis using the Antibiotic Resistance Genes Database (ARDB) (16) led to the identification of several genes (*marB, mecR, pbp2b, catB2, bacA, smeD,* and *oprM*) putatively involved in antibiotic resistance, some conferring specific resistance to macrolides, methicillin, penicillin, chloramphenicol, bacitracin, fluoroquinolone, and/or aminoglycosides.

The genome sequence and analysis of P. aquatile CR182^T^ in this study provide information to further explore and reveal the mechanisms of antimicrobial action in aquatic environments.

### Accession number(s).

This whole-genome shotgun project has been deposited at DDBJ/ENA/GenBank under the accession number POSP00000000 (BioProject number PRJNA429314). The version described in this paper is the first version.
